# Oligodendroglial Process Formation is Differentially Affected by Modulating the Intra- and Extracellular Cholesterol Content

**DOI:** 10.1007/s12031-012-9833-2

**Published:** 2012-06-28

**Authors:** Matthias Schmitz, Sandra C. Signore, Inga Zerr, Hans H. Althaus

**Affiliations:** 1 Max-Planck Institute of Experimental Medicine, RU Neural Regeneration, Hermann-Rein-Straße 3, 37075 Goettingen, Germany; 2 Department of Neurology, University Medicine Göttingen, Georg-August University Goettingen, Robert-Koch-Straße 40, 37075 Goettingen, Germany; 3 Department of Neurology, University Medicine Göttingen, Georg-August University Goettingen, Robert-Koch-Straße 40, 37075 Goettingen, Germany

**Keywords:** Caveolin-1, Caveolin containing rafts, Cholesterol, Nerve growth factor, Niemann–Pick disease type C1-Like 1, Oligodendrocytes, TrkA

## Abstract

Cholesterol is an essential component of eukaryotic plasma membranes and plays an important role in membrane organization and signaling processes. It is the major lipid component of detergent resistant caveolin-1 containing rafts which previously had been reported as a platform for nerve growth factor (NGF) signaling in oligodendrocytes (OL). Surprisingly, a knockdown of caveolin-1 attenuated the process formation of OL (Schmitz et al. J Neurosci Res 88:572–588, 2010), for which a loss of cholesterol could be responsible. In the present report, we could show that a caveolin-1 knockdown resulted in an elevation of cellular cholesterol level; it may indicate an important role of caveolin-1 in cholesterol trafficking to the plasma membrane. Treatment with exogenous PEG cholesterol, which was incorporated to the plasma membrane, supported oligodendroglial process formation, in particular when OL were stimulated by NGF. In this context we have found that OL express NPC1L1 (Niemann–Pick disease type C1-Like 1) which could modulate cholesterol uptake. In contrast, depletion of membrane-bound cholesterol diminished NGF-induced process formation concomitant with a reduced activity of p42/44 mitogen-activated protein kinases.

## Introduction

A noteworthy characteristic of myelin is that it contains an exceptionally high content of lipids (over 70 % of dry weight). More than 25 % of the total lipid content is cholesterol, which represents the largest proportion of lipid molecules when based on a molar ratio (Norton and Cammer 1984). Cholesterol plays an important role in myelination (Saher et al. 2005), dendrite differentiation (Goritz et al. 2005) and synaptic activity (Mauch et al. 2001). It is endogenously synthesized, since plasma lipoproteins cannot pass the blood–brain barrier (Björkhem and Meaney 2004). Glial cells produce up to 90 % of neural cholesterol. Hence glial cells are relevant mediators to cholesterol homeostasis in the CNS (Dietschy and Turley 2004). Astrocytes are suggested to support neurons with cholesterol (Nieweg et al. 2009). Oligodendrocytes (OL) are able to synthesize cholesterol by themselves, which is particularly important during myelinogenesis (Dietschy and Turley 2004), but they may be supported by astrocytes during this period.

In addition to these tasks, cholesterol can be detected in detergent-insoluble membrane microdomains (London and Brown 2000) such as caveolae. Their coat protein caveolin-1 binds cholesterol (Murata et al. 1995) and requires cholesterol for oligomerization (Monier et al. 1996). Caveolin-1 is also involved in cholesterol transport processes (Fielding and Fielding 2006).

Previous studies had already reported that growth factors such as platelet derived growth factor or epidermal growth factor may use these cholesterol-enriched plasma membrane microdomains or caveolin containing rafts (CCR) as platforms for signaling (Paratcha and Ibanez 2002; Abulrob et al. 2004; Pike 2005; Gielen et al. 2006). In particular, NGF-receptors have previously been found in detergent-resistant CCR of PC12 cells (Huang et al. 1999; Peiro et al. 2000). Recently, it had been shown that the TrkA/NGF signaling pathway, by which pig OL can modulate their process regeneration (Althaus et al. 1992, 1997; Althaus and Klöppner 2006; Althaus et al. 2008), and the de novo synthesis of myelin proteins (Althaus 2004) is modulated by CCR (Schmitz et al. 2010). These findings indicate that cholesterol serves not only as an essential structural element of the plasma membrane but it may also be involved in cellular signaling processes as a component of these platforms.

A lack of cholesterol results in flattening of caveolae (Matveev et al. 2001; Parpal et al. 2001; Dreja et al. 2002); it also promotes an internalization of signaling complexes (Chang et al. 1992; Furuchi and Anderson 1998; Prevostel et al. 2000) and a translocation of signaling complexes outside caveolae; under the latter condition, signaling processes were impaired (Peiro et al. 2000).

In the present study, we have investigated the role of cholesterol on porcine oligodendroglial process formation, since a caveolin-1 knockdown had attenuated oligodendroglial process formation via NGF (Schmitz et al. 2010), for which cholesterol imbalance might be responsible. We could show that a caveolin-1 knockdown resulted in an elevation of cellular cholesterol level. In contrast, an upregulation of caveolin-1 via NGF provoked a cholesterol decrease, indicating a role for caveolin-1 in cholesterol transport to the plasma membrane and in oligodendroglial cholesterol flux.

Treatment with exogenous cholesterol supported the formation of processes via NGF and the activation of p42/44 mitogen-activated protein kinases (MAPK) (Erk1 and 2). Rapid cholesterol depletion decreased NGF signaling, while moderate depletion of cholesterol via methyl-beta-cyclodextrin (MβCD) provoked an aggregation of OL.

In search for additional components, which may play a role in oligodendroglial cholesterol trafficking, we could detect the presence of the cholesterol transport protein NPC1L1 (Niemann–Pick disease type C1-Like 1), a component, which was as yet reported for intestinal absorption of cholesterol as well as a modulator for the caveolin transport and localization (Davies et al. 2005).

## Materials and Methods

All chemicals were of analytical grade where possible and obtained either from Sigma-Aldrich (Taufkirchen, Germany) or Merck (Darmstadt, Germany); culture media and fetal calf serum (FCS) were obtained from Biochrom (Berlin, Germany), Mezlocillin from Bayer (Leverkusen, Germany). ECL Western blotting detection kit came from Amersham (Freiburg, Germany).

### Cell Culture

OL were isolated from adult pig brains and cultivated on poly-d-lysine-coated Petri dishes or multiwell cultured plates by using an established protocol (Althaus et al. 1984; Bürgisser et al. 1988; Althaus and Klöppner 2006). Briefly, the white matter of mature pig (domestic, 6 months old) brains was dissected, minced, and sieved through nylon sieves of descending pore size. After that, cells were collected after centrifugation of the cell tissue suspension onto a discontinuous Percoll gradient, seeded and cultured on poly-d-lysine-coated Petri dishes or multiwall culture plates. The culturing protocol was as previously described (Althaus et al. 1991), except that the FCS in the culture medium was reduced to 5 %; transmission electron microscopy and immunocytochemical criteria identified the cells as mature (GC^+^, MBP^+^, PLP^+^, MOG^+^) OL (Althaus et al. 1991; Althaus and Siepl 1997; Althaus and Klöppner 2006); A2B5^+^, GFAP^+^, or OX4^2+^ cells were initially observed rarely if at all (Althaus and Siepl 1997); anti-MOSP IgM (Chemicon/Millipore, Schwalbach, Germany) diluted 1:1,000 was routinely used to specifically label OL (Dyer and Matthieu 1994) in this study.

### Morphometric Measurement of the Oligodendroglial Fibers

Morphometric evaluation of oligodendroglial process formation and process length occurred according to a previous protocol (Althaus et al. 1991). Briefly, phase contrast photographs were taken at random positions in the culture dishes (four different preparations). Photos were magnified to a suitable size. The determination of the oligodendroglial processes formation was performed by computing the average length of fibers per cell in arbitrary/relative units (r. U.) by a map measurer.

### Immunocytochemical Analysis

The following antibodies were used to visualize the indicated proteins: polyclonal anti-caveolin IgG (Transduction Laboratories, Lexington, USA) diluted 1:1,000; polyclonal anti-NPC1L1 (Novus Biologicals Corporation, Littleton, USA), diluted 1:500 for Western blot and 1:100 for immunocytochemistry; monoclonal anti-beta actin IgG (Abcam, Cambridge, UK) diluted 1:10,000; anti-MOSP IgM (Chemicon/Millipore) 1:500. Alexa Fluor 488 conjugated goat anti-mouse IgM and IgG were used as secondary antibodies (Molecular Probes/Invitrogen, Darmstadt, Germany) diluted 1:1,000; ECL anti-rabbit and anti-mouse IgG, horseradish peroxidase linked (Amersham, Freiburg, Germany) diluted 1:1,000.

For immunochemical staining cells were fixed in methanol/acetic acid (9:1) and treated with 0.1 % Triton X-100; antibody incubations took around 1 h at room temperature.

### Detergent-Free Enrichment of Caveolin-Containing Rafts

A slightly modified version of the sodium carbonate method of Song et al. (1996) was used to enrich CCR. Very briefly, 50 × 10^6^ porcine OL (8 DIV) were scraped into 2 mL sodium carbonate pH 11.0 and homogenized by an all-glass Dounce homogenizer. The cellular nuclei were removed by centrifugation at 1,000 × *g*. The homogenate was adjusted to 45 % sucrose/MBS buffer (25 mM MES and 0.15 M NaCl, pH 6.5) and placed at the bottom of a Beckman centrifuge tube; on top, a discontinuous 5–35 % sucrose gradient (4 mL of 35 % sucrose and 4 mL of 5 % sucrose in MBS containing 250 mM sodium carbonate) was formed. After centrifugation for 22 h at 39,000 rpm (SW 41 rotor, Beckman), 12 × 1 mL fractions were obtained, washed in MBS buffer and used for analysis. Afterwards, we determined the amount of caveolin-1 and cholesterol in CCR-enriched fractions 4-6 in comparison to non-CCR fractions 1–3 and 7–12 (Schmitz et al. 2010).

### Cholesterol Treatment and Depletion

To investigate the influence of cholesterol on the oligodendroglial process formation, we exposed 8 DIV OL to the water-soluble derivate polyethylene glycol-600 cholesterol (PEG-600-chol) (Sigma-Aldrich, Deisenhofen, Germany). The PEG-600-chol was added to the cell media in a concentration of 100 μg/mL for the indicated times, PEG-600 served as a control. For acute cholesterol depletion, cells were treated with methyl-beta-cyclodextrin (MβCD) (Sigma-Aldrich) (10 mM) for 45 min. Long-term experiments with a moderate cholesterol depletion were carried out by using MβCD in a concentration of 3 mM.

### Determination of Cholesterol

The cellular cholesterol level was measured by the Amplex Red Cholesterol Assay (Molecular Probes/Invitrogen) according to the manufacturer’s instructions and by using an established protocol (Amundson and Zhou 1999). This assay was conducted in a 96-well microplate using 100 μL of reaction volume per well. After extraction of the cellular cholesterol according the modified protocol from Gamble (Gamble et al. 1978), we dissolved the extracted cholesterol in 50 μL reaction buffer containing 0.5 M K_2_PO_4_, 0.25 M NaCl, 25 mM gallic acid, and 0.5 % Triton X-100. The enzyme reaction started after addition of 50 μL working solution, containing 300 μM Amplex Red reagents (10-acetyl-3.7-dihydroxyphenoxazin), 2 U/mL HRP, 2 U/mL cholesterol oxidase, and 2 U/mL cholesterol esterase. After incubated at 37 °C for 30 min, fluorescence intensities were measured using a fluorescence microplate reader.

### Immunocytochemical Imaging of Fluorescent-Conjugated Cholesterol

Fluorescein-polyethylenglycol-50 cholesterol (fPEG-chol), a kind gift of Dr. Kobayashi (RIKEN, Saitawa, Japan), was used to monitor cholesterol uptake. 8 DIV OL were treated with fPEG-chol (1 μM) for 15 min and 24 h. The uptake and internalization of fPEG-chol could be demonstrated by fluorescence microscopy.

### Western Blotting

OL were pre-treated with 1 mM sodium orthovanadate for 1 h, washed with PBS (phosphate-buffered saline) containing a mixture of inhibitors (1 mM sodium orthovanadate, 1 mM sodium fluoride, 1 mM phenylmethylsulfonyl fluoride, 1 μg/mL aprotinin, and 1 μg/mL leupeptide) and harvested by scraping on ice. Cell lysates were dissolved in 2 % SDS, containing all inhibitors, for 30 min, denaturated in sample buffer (containing mercaptoethanol) and heated at 95 °C for 2 min. Protein samples of equivalent protein content (20 μg/lane; protein determination according to Neuhoff et al. 1979) were separated by 10–15 % sodium dodecyl sulfate-polyacrylamide gel electrophoresis (SDS-PAGE) according to Laemmli (1970) and transferred to polyvinylidene difluoride (PVDF) membranes (Amersham). The PVDF membranes were incubated with 5 % dried milk in PBS and 0.1 % Tween-20 for 1 h at room temperature and afterwards probed with the antibodies of interest overnight at 4 °C. ECL antibodies from Amersham were utilized as secondary antibodies. The bands were visualized by the enhanced chemiluminescence (ECL) detection system according to the manufacturer’s instructions.

### In-Gel Mitogen-Activated Protein Kinase Assay

Cells were exposed to NGF for the times indicated. The cell lysates were separated by SDS-PAGE using a gel in which 10 % MBP (myelin basic protein) was incorporated; renatured MAPK (Erks) activity was detected following a modified protocol (Althaus et al. 1997) of (Virdee and Tolkovsky 1995). The gel was incubated in assay buffer for 60 min at 30 °C allowing the transfer of radioactive phosphor to myelin basic protein; not incorporated radioactivity was removed by washing the gel several times in 5 % TCA containing 1 % (*w*/*v*) tetrasodium pyrophosphate. Afterwards, the gel was dried overnight and subjected to autoradiography at −70 °C, using an RX-Fuji X-ray film.

### Transfection of Pig OL and Caveolin Knockdown

Our experiments were based on findings of Ge and Pachter (2004) who reported about a half-life of astroglial caveolin-1 in between 12 and 18 h, which makes attempts to knockdown caveolin-1 reasonable. Caveolin-1 siRNAs were synthesized by Qiagen (Hilden, Germany) based upon the sequence of porcine caveolin-1 (PubMed Accession-number AY490204) with all characteristics of siRNA targeting constructs. In preliminary experiments (Schmitz et al. 2010), a panel of transfection reagents was tested by using unspecific fluorescent siRNA: Lipofectamin (Invitrogen), Oligofectamin (Invitrogen), jetSI-Endo (Biomol, Hamburg, Germany), Gene Silencer (PQ-Lab Biotechnologies, Erlangen, Germany), and RNAiFect (Qiagen); efficient transfection and cell vitality were the decisive criteria in our hands, best results were finally obtained with 3–4 μL jetSIEndo/mL and caveolin-1 specific siRNA (40–60 nM) solved in 200 μL Gibco OptiMEM (Invitrogen); stable siRNA complexes were formed after 15–20 min and added to the culture medium (MEM, 2 % FCS) in which the cells were incubated for 5–8 h or overnight. Thereafter, the medium was replaced by fresh culture medium containing 5 % FCS.

### Signal Quantification and Statistical Analysis

The quantification of the staining intensities of all Western blots and MAPK in-gel kinase assays were performed by the densitometric software Scion Image. Using the statistic program Graph Pad Prism 4 the ratios of different samples were tested for significance utilizing the two-sided unpaired Student’s *t* test. All *p* values below 0.05 (*p < 0.05) are considered as significant. The standard error of the mean (SEM) was calculated to assess the variations between different samples under the same conditions and depicted as error bars. The error bars represent the SEM of at least three independent attempts.

## Results

### Uptake of Exogenous Cholesterol

Polyethylenglycol cholesteryl ethers are a unique group of non-ionic amphipathic cholesterol derivates. These compounds are soluble in water but retain many of the structural aspects of cholesterol (Ishitsuka et al. 2005). To visualize a cholesterol uptake in living cells, a fluorescein ester of PEG-chol that contains a fluorescein on the distal end of the PEG chain was used (Ishitsuka et al. 2005).

**Fig. 1:**
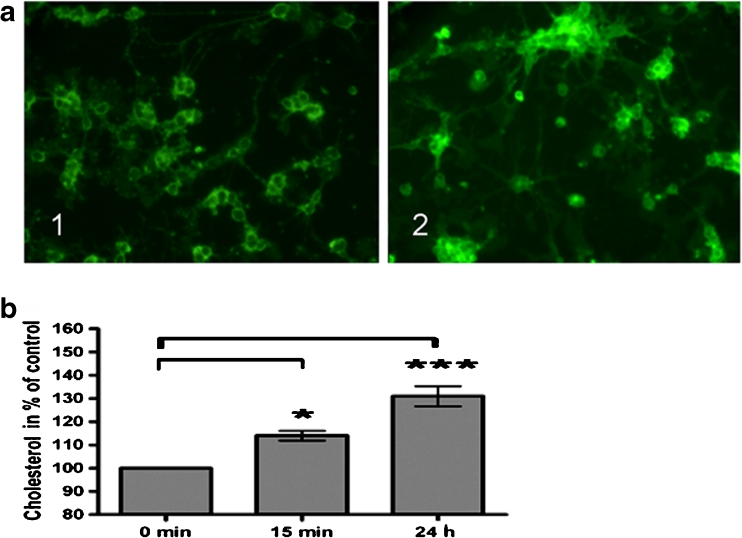
Uptake of PEG-chol in pig OL. **a** OL were exposed to fPEG-chol (1 μM). Portions of fPEG cholesterol distributed within the oligodendroglial plasma membrane after 15 min (*1*) and reached 24 h later cellular compartments (*2*), as demonstrated by immunofluorescence microscopy. **b** Extracellular exposure to PEG-600-chol (100 μg/mL) resulted in an approximately 10–15 % increase of total cholesterol level after 15 min and in a 30–35 % enhancement after 24 h. Quantification of total cellular cholesterol amount was performed by utilizing Amplex Red Cholesterol Assay. *P* < 0.05 was considered significant. Values are depicted as mean ± SEM

**Fig. 2:**
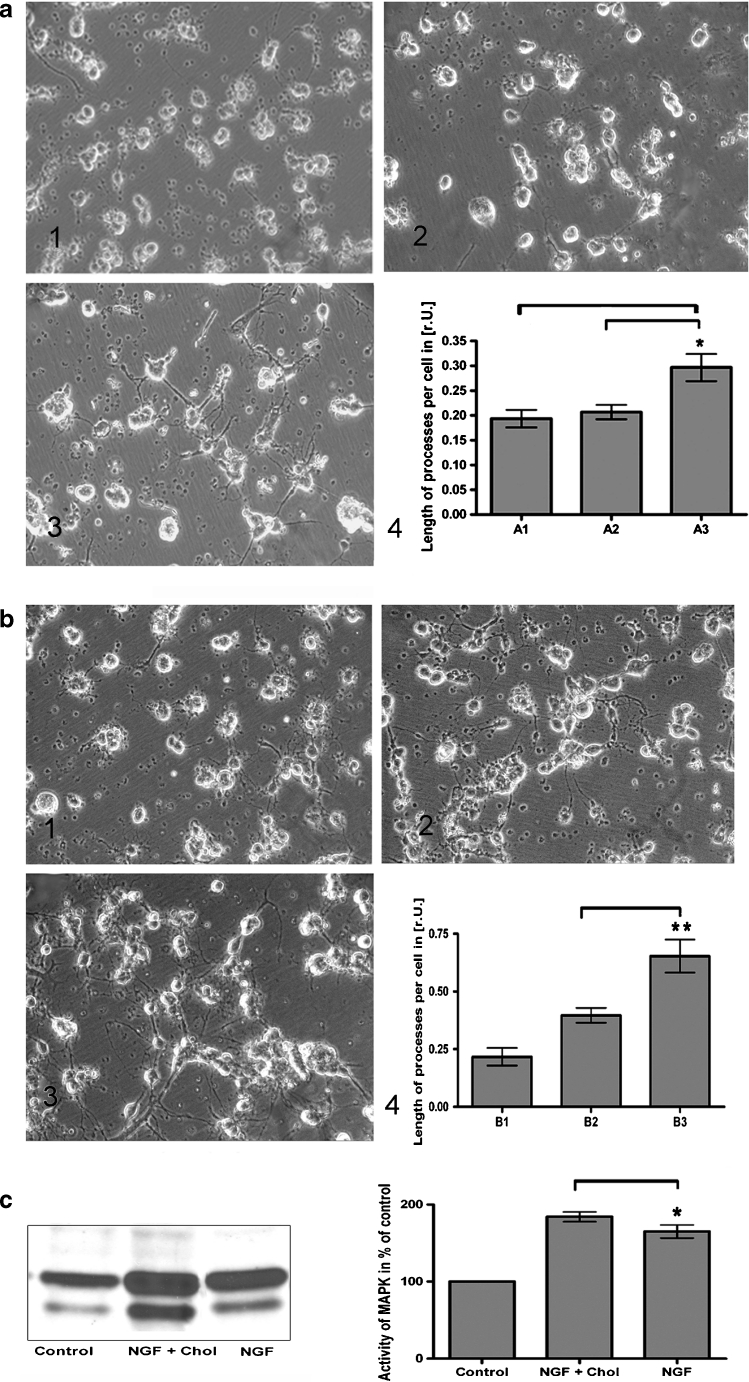
Effect of cholesterol on oligodendroglial process formation. **a** The morphometric evaluation of the oligodendroglial process formation (*4*) revealed that a treatment of OL (8 DIV) with PEG-600-chol (100 μg/mL) for 48 h (*3*) resulted in a significantly enhanced process formation compared to cells treated with PEG-600 (*2*), which behaved similar to untreated cells (*1*). **b** OL, preincubated with PEG-600-chol for 24 h, formed after 24 h NGF exposure significantly more processes (*3*), when compared to untreated control cells (*1*) or to cells treated with NGF for 24 h (*2*). An approximately twofold increase of process formation in NGF plus PEG-600-chol-treated cells was verified by morphometric evaluation (*4*). **c** An in-gel MAPK assay showed that the NGF-induced activation of MAPK (Erk1 and Erk2) after 4 h (*lane 3*) was significantly increased when cells were preincubated with cholesterol for 24 h (*lane 2*). *P* < 0.05 was considered significant. Values are depicted as mean ± SEM

Cells were incubated with fPEG-chol (1 μM) to monitor the dynamics of fPEG-chol uptake. Primarily oligodendroglial plasma membranes were stained; however, relative low amounts of fPEG-chol had already passed the plasma membrane after 15 min (Fig. 1a, 1); 24 h later, fPEG-chol was distributed to the plasma membrane and cellular compartments (Fig. 1a, 2). Supplementing the culture medium with exogenous PEG-600-chol (100 μg/mL) resulted in an increase of the cellular cholesterol level of approximately 10–15 % after 15 min and of 30–35 % after 24 h (Fig. 1b). A tendency to form aggregates of OL as reported for ascites tumor cells when the cells were enriched in cholesterol (Haeffner et al. 1984) could not be observed. PEG-600 alone did not affect the basic oligodendroglial cholesterol content of 10–20 μg/mg OL protein (Klopfleisch et al. 2008).

### Cholesterol, Exogenously Added, Promotes Oligodendroglial Process Formation and NGF Signaling

**Fig. 3:**
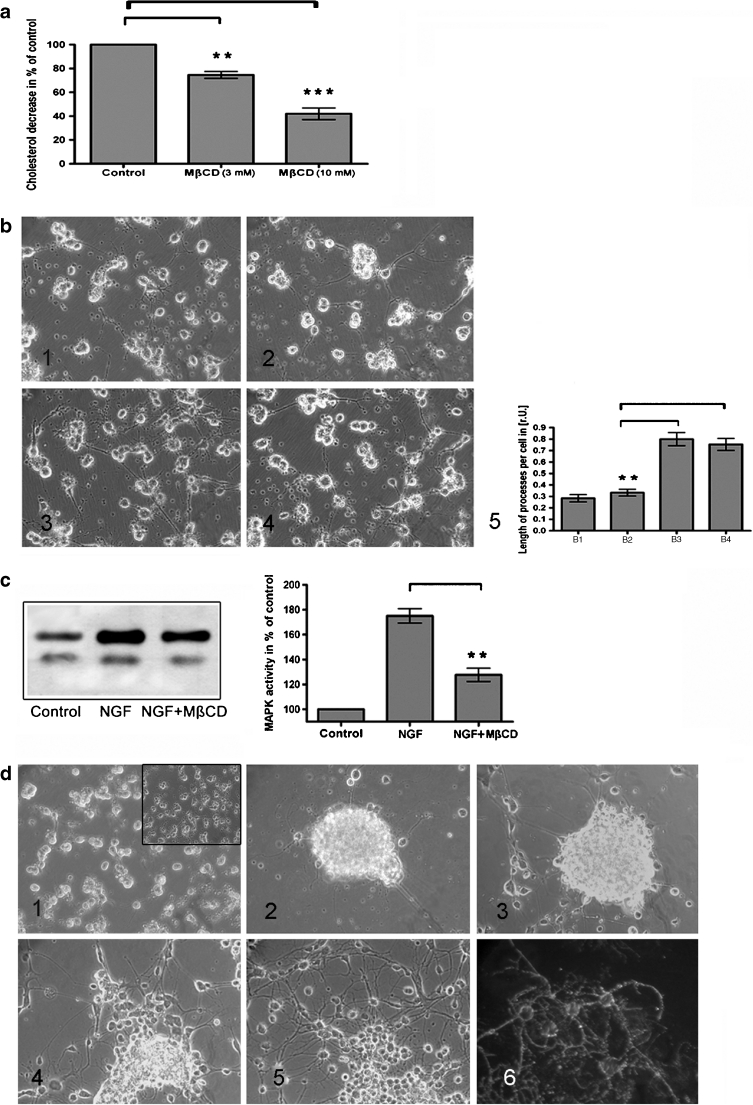
Depletion of cholesterol by MβCD. **a** Total cellular cholesterol levels in OL were decreased after incubation with MβCD (3 mM) for approximately 25 % and with MβCD (10 mM) for approximately 60 %. **b** Cholesterol deficiency provoked a significantly decreased process formation after NGF exposure for 48 h (*2*), compared to OL treated with NGF solely (*3*). The addition of cholesterol compensated this effect (*4*). Untreated OL showed a basal fiber generation (*1*). The morphometric evaluation revealed an approximately 2.5 to threefold decrease of process formation in cholesterol deficient cells after NGF exposure (*5*). **c** An in-gel MAPK assay revealed that the NGF-induced activation of MAPK (Erk1 and Erk2) was reduced in cholesterol depleted OL, which were preincubated with MβCD (10 mM) for 45 min and afterwards exposed to NGF for 4 h, compared to cells exposed to NGF solely. **d** Long-term exposure of OL with MβCD (3 mM) caused a retraction of OL processes after 24 h (*1*) compared to untreated control cells (*1*, *inset*) and an aggregation after 72 h (*2*). After removing MβCD and addition of PEG-600-chol (100 μg/mL) cells migrated out of the aggregates (*3–5*). Immunostaining with anti-MOSP revealed that the migrated cells were still matured OL (*6*). *P* < 0.05 was considered significant. Values are depicted as mean ± SEM

OL (8 DIV) were exposed to PEG-600-chol (100 μg/mL) and PEG-600 (100 μg/mL). The length of processes per cell was determined (“Materials and Methods”). We observed that process formation of PEG-600-chol-treated cells was significantly increased after 48 h (Fig. 2a, 3) compared to PEG-600-treated cells (*p* < 0.05) (Fig. 2a, 2) of which the morphology was comparable to untreated cells (Fig. 2a, 1). PEG-600-chol exposure also did increase MAPK activity; however, not significantly (data not shown).

Next, we examined the impact of cholesterol in combination with NGF. A significant increase of the length of oligodendroglial processes could be observed in 8 DIV OL, treated with PEG-600-chol (100 μg/mL) for 48 h plus NGF (100 ng/mL) for the last 24 h (Fig. 2b, 3), compared to NGF-treated cells without additional cholesterol (Fig. 2b, 2). Untreated control cells showed a basal length of processes (Fig. 2b, 1).

The ability of PEG-600-chol to promote oligodendroglial process formation under NGF was underlined by an increase of the activity of MAPK (Erk1 and Erk2), which was significantly more activated when NGF was used in combination with PEG-600-chol as compared to NGF-treated and untreated cells (Fig. 2c).

### Cholesterol Depletion Affects the Oligodendroglial Process Formation and Induces Cell Aggregation

MβCD is a cholesterol-binding drug which efficiently removes cholesterol from the plasma membrane (Christian et al. 1997). A mild depletion of cholesterol using 3 mM MβCD reduced the cellular cholesterol content to approximately 75 % after 24 h compared to that of untreated control cells. A higher and rapid depletion of oligodendroglial cholesterol was achieved by a treatment with 10 mM MβCD for 45 min which resulted in a decrease of approximately 60 % cholesterol (Fig. 3a).

**Fig. 4:**
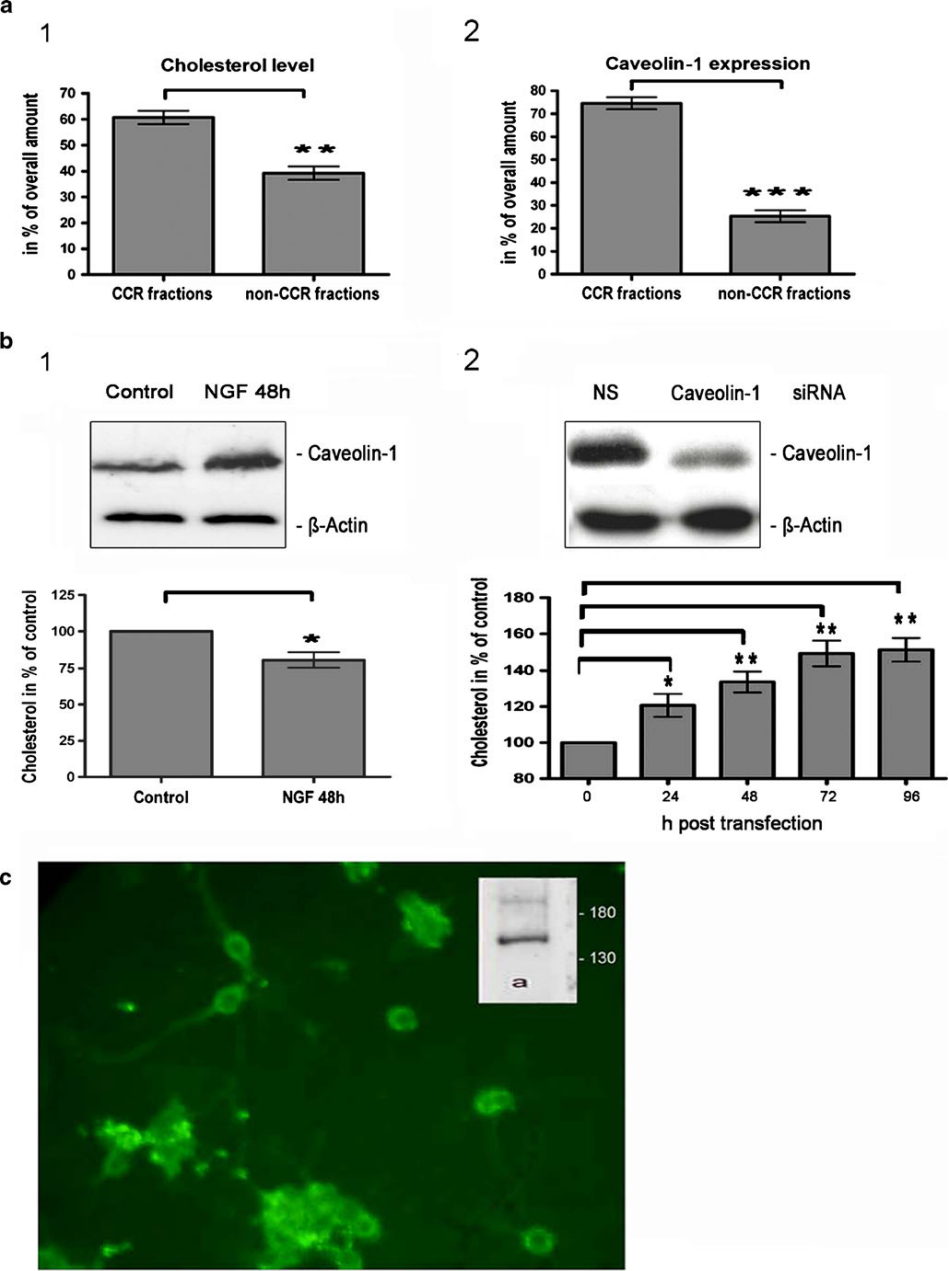
Influence of caveolin-1 on the cellular cholesterol level. **a** CCR were isolated according the sodium carbonate method of Song et al. (1996). Cholesterol content and caveolin-1 expression were determined; approximately 60 % of the cellular cholesterol and 75 % of caveolin-1 were located in the CCR-enriched fractions (*1*, *2*). **b** A treatment with NGF induced an upregulation of caveolin-1 expression after 48 h, shown by Western blotting (*1*, *inset*), while the cellular cholesterol content decreased simultaneously (*1*). Knockdown of caveolin-1 gene expression was demonstrated by Western blotting 48 h post-transfection (*2*, *inset*). The oligodendroglial cholesterol content was significantly enhanced 24 and 96 h post-transfection (*2*). **c** Immunostaining with anti-NPC1L1 and Western blotting (*inset*, *lane a*: NPC1L1) revealed the expression of this cholesterol transport protein in pig OL. *P* < 0.05 was considered significant. Values are depicted as mean ± SEM

Rapid cholesterol depletion affected the NGF-induced process formation. Pretreatment of OL with MβCD (10 mM) for 45 min impaired the NGF-induced oligodendroglial process formation after 48 h markedly (Fig. 3b, 2). Cells solely stimulated with NGF showed the expected NGF effect (Fig. 3b, 3). The addition of PEG-600-chol to the NGF-containing medium could compensate the cholesterol depletion (Fig. 3b, 4). Untreated control OL developed a basic number of short processes during this time period (Fig. 3b, 1).

In this context, we analyzed the effect of an acute cholesterol depletion on downstream components of the NGF signaling cascade. We could show that a cholesterol deficiency did not only affect the morphological appearance of OL but also the NGF signaling cascade: the activation of p42/44 MAPK (Erk1 and Erk2) was significantly reduced after NGF exposure for 4 h compared to untreated cells (Fig. 3c).

While almost all experiments with MβCD use higher concentrations of MβCD in a relative short time, we were interested to know how OL react on moderate cholesterol depletion within a prolonged time frame. We incubated OL with 3 mM MβCD for 72 h. A decreased level of cholesterol of approximately 25 % induced a retraction of oligodendroglial processes within 24 h (Fig. 3d, 1); 72 h later an aggregation of the cells has occurred (Fig. 3d, 2). Addition of PEG-600-chol (plus removal of MβCD) dispersed oligodendroglial aggregates; after 24 h (Fig. 3d, 3) to 72 h (Fig. 3d, 4), some cells detached from each other, 7 days later the aggregates were completely dispersed (Fig. 3d, 5). Immunostaining with anti-MOSP, an oligodendroglial specific marker (Dyer and Matthieu 1994), demonstrated that the cells, released from the aggregates, were still mature OL (Fig. 3d, 6). In contrast, OL started to detach from the dish when MβCD was removed without adding PEG-600-chol.

### Role of Caveolin-1 Knockdown on Cholesterol Homeostasis

Since caveolin-1 acts also as a cholesterol transport protein (Fielding et al. 1999), we were interested to know as to whether a knockdown of oligodendroglial caveolin-1 (Schmitz et al. 2010) might affect the cholesterol content in pig OL. Caveolin-enriched membrane fractions were isolated by utilizing the sodium carbonate method of Song et al. (1996). We pooled the caveolin-enriched fractions (4–6) versus the remaining fractions (fractions 1–3; 7–12) and determined the cholesterol level as well as the caveolin-1 expression. Approximately 60–65 % of the cellular cholesterol and 70–75 % of the amount of caveolin-1 were located in the caveolin-enriched fractions (Fig. 4a, 1 and 2). Treatment with NGF resulted in a marked upregulation of caveolin-1 expression after 48 h (Fig. 4b; 1, inset) and as shown previously (Schmitz et al. 2010). At the same time point, we observed that the cellular cholesterol amount was reduced significantly (Fig. 4b, 1).

Specific short interference RNAs (siRNA) were a valuable tool to downregulate the expression of caveolin-1. An effective knockdown of caveolin-1 after 48 h was shown by Western blotting (Fig. 4b; 2, inset); β-actin served as an internal control. Cells, treated with non-specific (NS)-siRNA under the same conditions, expressed caveolin-1 (Fig. 4b, 2, inset) similar to untreated OL (data not shown).

Interestingly, a caveolin-1 knockdown induced a significant enhancement of the cellular cholesterol content 24–96 h post-transfection (Fig. 4b, 2).

In this context, we could show for the first time that NPC1L1 (Niemann–Pick C1-Like 1, MW ~145 kDa), known for serving as a plasma membrane anchored cholesterol sensing receptor in the intestine (Better and Yu 2010; Davies et al. 2005), is present in pig OL (Fig. 4c, inset). It seems to be localized to the plasma membrane and to intracellular compartments as revealed by immunolabeling (Fig. 4c).

## Discussion

Although much knowledge has accumulated concerning the regulation of cholesterol in the human body, only less information is available about the role of oligodendroglial cholesterol. OL play an essential role in myelin biogenesis (Saher and Simons 2010); cholesterol is an important compound of the resulting myelin sheaths.

### Role of Cholesterol in Oligodendroglial Signal Transduction

In our study, we were interested to know as to whether cholesterol participates in oligodendroglial NGF/TrkA signaling which affects the myelination process (Althaus et al. 1997). Treatment of pig OL with external cholesterol derivates revealed that the cells were able to incorporate cholesterol into the plasma membrane followed by a slow cellular distribution; a course already reported for other cells (Sato et al. 2004). We observed that an increase of the oligodendroglial membrane cholesterol content induced an enhanced process formation, which was even more accelerated when the cells were exposed to NGF for 24 h. This finding indicates that the actual cellular cholesterol level affects the NGF signaling cascade.

A cellular fractionation according to the sodium carbonate method (Song et al. 1996) showed that most of the oligodendroglial cholesterol (~60 %) and caveolin-1 (~75 %) could be detected in the CCR-enriched fractions in addition to TrkA and MAPK (Schmitz et al. 2010). These data suggest that cholesterol may be involved in the oligodendroglial signaling by regulating the composition of CCR, which had been described previously to play a modulating role during the oligodendroglial process formation via NGF (Schmitz et al. 2010, 2011). Cholesterol is important for the function and organization of membrane proteins and receptors (Pucadyil and Chattopadhyay 2006). This effect could either be due to a specific molecular interaction between cholesterol and signaling molecules or due to alterations of the membrane fluidity (Kwik et al. 2003; Byfield et al. 2004). The residue 94–101 within the caveolin scaffolding domain (82–101) contains a cholesterol recognition sequence that binds free cholesterol (Epand et al. 2005). The binding of cholesterol to caveolin plays a relevant structural and functional role in CCR (Pike 2005; Thomas and Smart 2008). Structurally, it is needed as a stabilizing structure element of lipid rafts or CCR and to maintain the invaginated form of caveolae. Functionally, cholesterol may facilitate the ability of some proteins to associate with CCR and to form the basis for specific clustering events which is responsible for signal transduction and membrane trafficking (Brown and London 1998; Incardona and Eaton 2000). Moreover, cholesterol exposure may result in an increase in cell-surface caveolae by induction of caveolin-1 synthesis (Fielding et al. 1997; Bist et al. 1997) or an enhanced transfer of caveolin-1 from cellular pools to CCR (Thyberg 2002).

### Moderate Cholesterol Depletion Induces Aggregation of OL

The pharmaceutical use of cyclodextrins has been discussed in several articles (Rao and Stella 2003; Loftsson and Brewster 2010). Cyclodextrins enhance the solubility and chemical stability of drugs by forming a 1:1 to 2:1 inclusion complex (Nishijo et al. 2003; Stella and He 2008); the on/off rates of the equilibrium binding depend on the dilution of the complex (Stella and He 2008). Of the various cyclodextrins present, MβCD is a useful tool to investigate the function of cholesterol. It sequesters cholesterol in its hydrophobic pocket (Christian et al. 1997) and efficiently removes cholesterol from the plasma membrane which hosts ca. 75 % of the total unesterified cellular cholesterol (Liscum and Munn 1999). Keratinocytes exposed to higher concentrations of MβCD (>50 % cholesterol depletion) for a longer period of time (24 h) undergo a substantial cell death probably by inducing an apoptotic pathway via caspases (Schönfelder et al. 2006; Mahammad et al. 2010). Pig OL were tolerant to a mild depletion of cholesterol (25 %) via MβCD over 3 days. OL retracted their processes and aggregated but remained attached to the Petri dish. However, after removal of MβCD and addition of cholesterol to the medium the cell clumps dispersed. An oligodendroglial-specific labeling showed that the cells present were still OL; a substantial cell death has not occurred. The observed cell aggregation has probably been attributed to the cholesterol depletion and not to an MβCD effect per se. This assumption is supported by the finding that OL start to detach from the Petri dish after simple removal of MβCD from the cell medium without replenishing cholesterol. Similarly, in a study on T cells, where limited cholesterol depletion caused an aggregation of lipid rafts, an unspecific effect of MβCD could be excluded (Mahammad et al. 2010). Cholesterol plays a crucial role in plasma membrane organization (Pucadyil and Chattopadhyay 2006). Hence, a cholesterol depletion can result in a plasma membrane reorganization, aggregation of lipid rafts, altered growth factor receptor function, enhanced protein expression or suppressed cell migration depending on the cell type investigated (Westover et al. 2003; Jeon et al. 2010; Mahammad et al. 2010). Which of these effects are finally involved in the observed aggregation of OL remains to be investigated.

### Rapid Depletion of Cholesterol Impaired NGF Signaling

The depletion of cholesterol from the plasma membrane by MβCD disrupts caveolae and interferes with their functionality (Hailstones et al. 1998; Thorn et al. 2003).

Pig OL pre-treated with 10 mM MβCD for 45 min showed that a lack of cholesterol provoked a compromised oligodendroglial process formation via NGF as well as an inhibition of downstream components of the NGF signaling such as p42/44 MAPK activity. In PC12 cells, a depletion of membrane cholesterol decreased the magnitude and duration of NGF-stimulated p42/44 MAPK as well as the phosphorylation of TrkA (Peiro et al. 2000; Limpert et al. 2007). Repletion of cholesterol in PC12 cells restored their ability to activate MAPK upon NGF stimulation (Peiro et al. 2000). Previous results on OL treated with simvastatin, an inhibitor of the cholesterol synthesis, revealed an impairment of myelin components in vitro and a retardation of ongoing remyelination in vivo (Klopfleisch et al. 2008). This effect was in part attributable to a diminished prenylation of small G-proteins but also to a cholesterol depletion indicating a central role of cholesterol for myelination (Saher and Simons 2010).

A reason for these findings might be that cholesterol is involved in the formation of rafts/caveolae. Depletion of cholesterol from caveola-rich membranes, which are linked to the cytoskeleton (Stahlhut and van Deurs 2000), leads to increased membrane stiffness (Kwik et al. 2003; Byfield et al. 2004), a functional disruption of lipid rafts (Thorn et al. 2003) with a dramatic change of the membrane raft proteome (Zheng et al. 2011), caused by a dissociation of signaling proteins from caveolae, a disassembly of caveolin multimeres (Westermann et al. 2005) and flattening of caveolae (Westermann et al. 2005; Fielding and Fielding 2006; Thomas and Smart 2008). Signaling proteins moved to non-raft regions of the plasma membrane, where signaling transmission may be less efficient (Pike 2005).

### Downregulation of Caveolin-1 Increases the Oligodendroglial Cholesterol Level

A direct interaction of caveolin-1 and cholesterol had been described by Murata and colleagues (Murata et al. 1995). However, only little is known about the regulation of the cholesterol homeostasis in OL and their function for the oligodendroglial process formation. In particular, the synthesis, the cellular trafficking, uptake, and efflux are not yet understood completely despite the fact that OL are, besides astrocytes, the major cholesterol-producing cells in the CNS.

In our study, we found that a knockdown of caveolin-1 resulted in an elevation of cellular cholesterol level. However, this increased cholesterol level was concomitant with a decline of oligodendroglial process formation whether or not OL were exposed to NGF (Schmitz et al. 2010). A rational explanation of these apparently contradictory data would be that caveolin-1 plays a crucial role for the intracellular trafficking of cholesterol. The transport of newly synthesized cholesterol to the plasma membrane may be impaired by the low caveolin-1 level. Depletion of cholesterol from the plasma membrane attenuates cellular signaling (Pike 2005). The exact mechanism by which cholesterol efflux and cholesterol transport to the plasma membrane is regulated is as yet not completely understood.

Previous studies had already suggested a role of caveolin-1 and CCR in maintaining the cellular cholesterol balance (Roy et al. 1999; Ikonen and Parton 2000; Fielding and Fielding 2000). Caveolin-1 has been found to play a crucial role in non-vesicular cholesterol trafficking. In particular, it may be involved in the trafficking of newly synthesized cholesterol from the ER to the plasma membrane (Smart et al. 1996; Incardona and Eaton 2000) which would support our assumption. Caveolin-1 might form a kind of chaperon complex (Uittenbogaard et al. 1998). This complex could already be isolated. It comprised of cholesterol, caveolin-1, cyclophilin (cyp) A, cyp 40, and heat shock protein 56 (Uittenbogaard et al. 1998).

Other findings indicate a role of CCR in cholesterol transport. Relevant cholesterol transport proteins such as LDLR-ApER-2 or the scavenger receptor class B type1 (SR-BI) are associated with CCR (Babitt et al. 1997; Graf et al. 1999; Matveev et al. 1999; Frank et al. 2002). Overexpression of SR-BI and caveolin-1 significantly increased cholesterol efflux in other cells (Truong et al. 2010). In addition, ATP-binding cassette transporters, which can regulate the transport and efflux of cholesterol across the plasma membrane, are located in CCR (Mendez et al. 2001; Jodoin et al. 2003). However, it remains to be shown which of these transport pathways exist in pig OL.

Interestingly, we could show that another potential “team player” in cholesterol homeostasis is expressed in OL: NPC1L1, a protein involved in cholesterol uptake and transport (Altmann et al. 2004; Betters and Yu 2010). Its putative MW of ~145 kDa as shown by Western blotting and its immunocytochemical localization correspond to results for other tissues (Davies et al. 2005; Temel et al. 2007; Yu et al. 2006). Studies on hepatoma cells have revealed that NPC1L1 is predominantly localized to intracellular components but relocated to the plasma membrane when acute cholesterol depletion via MβCD occurred (Yu et al. 2006). The NPC1L1-mediated cholesterol uptake seems to occur via a cholesterol-regulated clathrin-dependent endocytosis (Betters and Yu 2010; Yu et al. 2006). NPC1L1 mRNA has been found with highest values in the intestine with variant expression in rodent and human liver indicating species differences; other tissues such as the lung or brain also expressed NPC1L1 but at a relatively low level (Davies et al. 2000, 2005; Pramfalk et al. 2011).

The presence of NPC1L1 in OL is remarkable for the following reasons: (1) It would be of interest to know as to whether NPC1L1 is upregulated during myelinogenesis, a period of highest cholesterol demand since cholesterol synthesis requires substantial energy input (Temel et al. 2007); (2) NPC1L1 is regulated via SREBP2 (Sterol Regulatory Element Binding Protein) which is linked to the MAPK cascade (Kotzka et al. 2000; Pramfalk et al. 2011); MAPK in turn are stimulated by various growth factors active during oligodendroglial maturation and subsequent myelinogenesis; (3) it was shown in a previous report that a lack of NPC1L1 activity causes a deregulation of caveolin transport and localization (Davies et al. 2005). Could NPC1L1 also affect the function of caveolin in OL? Having in mind that caveolin containing rafts are important for growth factor signaling (Schmitz et al. 2010) it seems to be a point necessary to be answered.

In conclusion, the present study provides evidence that cholesterol plays an important role in oligodendroglial process formation and signal transduction ± NGF. Our caveolin-1 knockdown data indicate a crucial involvement of caveolin-1 in oligodendroglial cellular cholesterol trafficking.

## Abbreviations

CCR: caveolin containing rafts
DIV: days in vitro
siRNA: small interfering RNA
MAPK: mitogen-activated protein kinases
MβCD: methyl-beta-cyclodextrin
NGF: nerve growth factor
NPC1L1: Niemann–Pick disease type C1-Like 1
OL: oligodendrocytes
PEG-600-chol: polyethylene glycol cholesterol
TrkA: tyrosine kinase A
